# Hospital and Institutional News

**Published:** 1915-08-07

**Authors:** 


### HOSPITAL AND INSTITUTIONAL NEWS.

### IN ST. PAUL'S CATHEDRAL, AUGUST 4, 1915.

One year of the great war has gone, and on the 4th inst. at noon the first anniversary service of humble prayer to Almighty God on behalf of the nation and Empire was held in St. Paul's. Once again the crowded congregation, the impressive service, and the imposing architecture of this wonderful creation of Wren's made many present, who have seen both cathedrals, realise how far St. Paul's, as it is to-day, exceeds in dignity and force St. Peter's at Rome. The many thousands present, representing all classes of people, from T.M. the King and Queen to the humblest of their subjects, were united in prayer, for everyone present entered so heartily into the proceedings that the unison was perfect. The Archbishop of Canterbury began his sermon with the words, "Watch ye, stand fast in faith, quit you like men, be strong," and each person present seemed to take the words to heart and to embrace them as watchwords for future conduct and action. Members of the medical and nursing profession, with the men of both Services present, felt the depth of meaning the words conveyed. They, indeed, thanked God for the comfort they have proved to be in many a trying period of the war already. It was noticeable with what skill and continuous watchful care the nurses in charge of wounded soldiers guarded them from pain and danger arising from the crowding of so great a congregation on leaving the cathedral. Everything was arranged admirably. The full band of the Royal Artillery was inspiring, and its introduction to the National Anthem stirred all hearts, and brought tears into many eyes. The Archbishop properly and forcibly reminded the congregation that the war was none of our seeking, for we laboured hard and continuously to preserve peace. So having weighed fully the right and wrong of the matters in dispute, we could commend the cause of the Allies with a clear conscience to the protection and guidance of God. He reminded every man and woman throughout the Empire that the well-being of the world for future centuries may hang upon our action and decision in the conduct and continuance of the war, until right is victorious and wrong redressed to the full. This is a great heritage and responsibility for England, and for every man and woman amongst us. Watch ye, stand fast in faith, pray continuously. It was a remarkable service which put heart and strength into everyone present. Its influence will spread and bear fruit throughout the British Empire, and amongst our Allies too. Quit you like men! Be strong!

### THE SUCCESS OF HOSPITAL SUNDAY.

The main contention contained in the Special Appeal Number of The Hospital, which this year, as usual, was published in connection with the anniversary of Hospital Sunday, was the confident prediction that this year the Sunday Fund would produce a record. Our prophecy has been amply fulfilled. We give on another page a full report of the council meeting of the Metropolitan Hospital Sunday Fund at the Mansion House, and a full list of the awards. The main fact of public interest announced at the meeting was that the total of the Fund amounted to £70,600, which is £11,000 more than the total sum raised this time last year. Two points of interest in the speeches may be touched on here. The first was made by Sir Ernest Tritton, who presided at the council meeting. He pointed out that the report of the Distribution Committee proved that the population of London was more anxious than ever to do their best on Hospital Sunday, because they realised the magnificent work which the voluntary hospitals were doing for the wounded. This fact, it will be remembered, was the ground we gave for our belief that the total sum raised this year would be worthy of the occasion. It is not unbecoming, also, to quote Lord Meath's tribute to the newspaper as the means without which it would be impossible to raise the immense sums that are now obtainable for charity. In pursuance of the policy that a thoroughly interested, or, in other words, a well-informed, public is the best guarantee for voluntary hospitals' financial support. So we have maintained our Special Appeal Number on Hospital Sunday, which gives a bird's-eye view of the work done in such a way also as to make that work appeal to the eye and imagination of the layman previously uninstructed in the subject. We rejoice in the £70,600 raised this year. We regard it as a good augury and a just tribute to Sir Ernest Tritton's unceasing devotion to this Fund.

### THE NEW ZEALAND HOSPITAL AT WALTON-ON-THAMES.

New Zealand, which has played a part as much deserving of our gratitude and praise as that of any other of the great Dependencies, is following the example set by Canada and Australia in establishing a hospital for her wounded sons. In view of the fine achievement of the New Zealand contingent in the Dardanelles the new institution will be especially welcomed, and Walton-on-Thames has been chosen for its situation. Thanks to private generosity a house and grounds have been rented, and 150 beds or more will be eventually established. The undertaking has been brought about by co-operation between the New Zealand War Contingent Association, the Government, and the War Office. The first batch of patients arrived last week, and it is significant of the existing need which has been felt for a New Zealand Hospital that the men forming the earliest patients in the new hospital are drawn mainly from scattered institutions throughout the country. In the absence of a hospital for New Zealanders, it was, of course, necessary that the wounded men of the

New Zealand contingent should, at all events in the first place, be drafted to whatever hospital was available. But the war, which has knit closer than ever the existing bonds of the English-speaking race, has also served to demonstrate the loyalty felt by all patriotic men to the soil which bred them and to the actual country of their birth. Such a home atmosphere in the Mother-country is provided for New Zealanders by the new institution at Walton-on-Thames. We have no doubt that the appeal of the Thames will be all the more penetrating from the fact that New Zealanders are tended by their fellow-countrymen in a hospital reserved for themselves. At the opening last Saturday the High Commissioner for New Zealand, the Hon. Thomas Mackenzie, announced that the cost of equipping and maintaining the hospital would be from £15,000 to £20,000. In this country a fund has been raised, which amounts to some £9,000, for the assistance of New Zealand soldiers, although it is understood that no appeal has been made to the British public. At present 110 patients can be accommodated. The King is expected to pay a visit to the institution.

### DANGERS TO ATTENDANTS IN MENTAL WARDS.

The dangers to which attendants in mental wards in the Metropolitan institutions may be liable was exemplified in a case which has occurred at St. George's Institution in the Borough, which is under the control of the Southwark Board of Guardians. A male nurse called upon an inmate to assist in restraining a patient in the mental ward, when the patient, a one-legged man, kicked the inmate, who afterwards died. He was, however, suffering from chronic Bright's disease, and the medical evidence showed that the man died from syncope from that disease, which was probably accelerated from shock consequent upon the injury. The case shows that it is most inadvisable for Poor-Law authorities to be dependent on the inmates, and consequently upon untrained men, in cases of this kind, though there is always the question of keeping a large staff in waiting for exceptional emergencies. With the shortage of male nurses and the increased salaries which the authorities are called upon to pay, it would appear as if at the present time the Boards of Guardians may have to be satisfied with what service they can get. The Board have under consideration the question of these mental cases, and it is to be trusted that their deliberations will result in the gradual exclusion of untrained helpers.

### MR. LLOYD GEORGE AND WAR SERVICE BADGES.

In view of the interest aroused in the subject of War Service Badges among members of hospital staffs, and of the illustrated articles which we have published on this matter, it is interesting to give the following quotation from the *London Gazette* of Friday last week: "Notice is hereby given, under the Rules Publication Act, 1893, that it is proposed by the Minister of Munitions, after the expiration of at least forty days from this date, in pursuance of the powers conferred upon him by Section 8 of the Munitions of War Act, 1915, and every other power enabling him on that behalf, to issue Rules as to War Service Badges in England and Ireland. Draft copies of the said Rules can be obtained in the interval at the office of the Minister of Munitions." By the middle of September, therefore, the Rules on the subject may be expected to be in force, and in the meantime hospital managers may be interested to consult their provisions.

### SIX WEEKS IN A GERMAN INTERNMENT CAMP.

Dr. W. W. Kaye Brown, son of Dr. R. Kaye Brown, medical officer of health for the Borough of Bermondsey, has returned to London after six weeks' detention in the German internment camp at Heidelberg. Dr. W. W. Kaye Brown was a student at St. Thomas's Hospital. When the war broke out he at once volunteered for service, securing the appointment of probationer surgeon on the torpedo-boat destroyer *Maori*. When that vessel was unfortunately lost, Dr. Brown, along with the officers and crew, was taken prisoner by the Germans. He was placed in the camp at Heidelberg, which he describes as barracks, where he had as companions a number of Russian, French, and a few Belgian officers, with one Indian. The prisoners were well treated, and had plenty to eat and drink, being allowed sixty marks a month for messing. This was sufficient to cover the cost of living, but he and his companions had to appeal to their relatives for assistance in order to provide clothes and the little luxuries that are essential to life even in a German internment camp. He lost his kit when the *Maori* went down, and until he received help was left only with the clothes he wore. His leisure time was occupied in teaching English to his fellow-prisoners, who in return initiated him into the mysteries of French and Russian. He was released with a number of French officers, arriving in London little, if any, the worse for his incarceration in the German internment camp at Heidelberg.

### LOCAL CONGRESSES AND GIFTS TO HOSPITALS.

A good example of the maxim "Great things are done by the help of small ones" is recorded in a gift of £70 which has been made to the Leicester Royal Infirmary to be used for a specific purpose to be selected by the board of governors. The gift arises from the Whit-week Congress of the Co-operative movement held in Leicester. A good many years ago Messrs. Blandford and Henry Clarke accepted the self-imposed task of collecting from the Co-operators attending the annual Congress voluntary contributions to be used for some good charitable purpose in the town in which the Congress was held. The effort resulted in many struggling institutions receiving substantial support. The movement continued a voluntary one for some years, until officially it was decided to make a levy of 1s. per member attending the Conference and hand the result to the local executive for distribution to a local charity. The outcome of the Leicester Conference is seen in the gift of £70 above recorded. In this way, year by year, a substantial sum has been distributed by the Co-operative delegates, which is in keeping with the contributions made by the Wholesale Co-operative Society year by year to hospitals and charities. We hope the achievement of the Co-operative Congress will be emulated at all congresses, whether secular or otherwise, and we bring the idea to the notice of hospital superintendents as one which is well worth their attention when conferences are arranged in their locality.

### LONDON INSURANCE COMMITTEE'S PHARMACOPŒIA.

The London Insurance Committee have issued to all medical practitioners and chemists on the panel a copy of the "London Insurance Pharmacopœia, 1915." This has been compiled by the Panel Committee for the County of London, in conference with the Pharmaceutical Committee and two professional members of the London Insurance Committee. It is pointed out by the compilers, in the preface, that it is not intended to restrict the character of the medical treatment to insured persons; the idea is only to facilitate the work of the panel doctor. A useful list of the more important alterations in strength and dose of preparations in the "B.P. 1914" is incorporated in the notes, together with approximate metric and imperial equivalents of weights and measures. A list of changes in nomenclature appears on page 4; thus "Hexamine" now appears as the official name for Hexamethylenetetramine or Urotropia. Considerations of economy appear to have weighed with the compilers; thus we find, instead of "spirit of chloroform," an emulsion made with soap-bark tincture is used in many formulæ. Liquid extracts nux vomica and cinchona replace the more often prescribed tinctures. This, of course, follows on the general procedure in economising at hospital dispensaries. It should be remembered, however, that in certain cases the stimulant effect of alcohol (as in sal volatile) is by no means negligible, so that the use of "aqueous" tinctures, etc., without regard to the action desired, is to be deprecated. On the whole, this little book may prove a useful adjunct to treatment of insured persons by the busy panel practitioner, although independent medical men will still, no doubt, continue to use their favourite formulæ in prescribing for their insured patients.

### A PRACTITIONER'S SUICIDE WITH VERONAL.

At an inquest held at Chelsea Town Hall on the body of a medical practitioner, Dr. Malcolm O. Cruickshank, who was discovered, with the housekeeper, lying unconscious in the sitting-room of the flat above his surgery in Chelsea, a verdict of "suicide while of unsound mind caused by domestic worry" was returned. Dr. Spilsbury, pathologist at St. Mary's Hospital, said that his *post-mortem* examination showed that, in his opinion, death was due to syncope from acute bronchial pneumonia, consequent upon narcotic poisoning. Evidence was given to the effect that the housekeeper had obtained, on a letter signed by Dr. Cruickshank, two separate half-ounces of veronal from a chemist. It was also stated that the deceased had been much worried owing to a disagreement with his wife, who was a Frenchwoman. He had told one witness, his sister, that he hoped that he would be killed at the Front. He had joined the Royal Army Medical Corps. The housekeeper, who recovered, and was present in court, stated that the intention of the doctor and herself was "to do it together." The housekeeper was arrested on the charge of attempted suicide.

### AUSTRALIA AND HER WOUNDED.

It is only fitting that the occasion of Australia Day, which was celebrated in the Commonwealth on July 30, should lead us to pay the deserved tribute due to the work which has been performed on behalf of the Australian wounded by their fellow-countrymen. Writing from Sydney, the *Times* correspondent notes that in New South Wales alone £160,000 in round figures has been collected on behalf of the men, and that each small township has vied with the others in generosity. A record is said to have been achieved in Sydney, where the chief feature in the celebrations, in which a simultaneous collection was held throughout the State, was a military procession, headed by a number of wounded from the Dardanelles. By 6 p.m., the writer states, a quarter of a million had been collected, including a few county returns. The grand total for the State is anticipated to be not far short of £500,000. This magnificent response, and the enthusiasm with which it was accompanied, not only pay a worthy tribute to the heroism of the Australian contingent, but provide a striking instance of the grip of the voluntary system of charitable support, even in portions of the Empire in which the voluntary principle is not wholly relied on for the maintenance of hospitals.

### ASSISTANT MEDICAL SUPERINTENDENTS' SALARIES INCREASED.

The North Infirmary Visiting Committee of the St. Pancras Guardians have recommended that the present salaries of the assistant medical officers should be increased. The proposal, which has received the Board's sanction, is that the senior assistant medical superintendent's salary be increased from £175 to £200 per annum, with board and residence, and that the salary of the junior assistant medical superintendent be £175, instead of £140 as at present. In view of the experiences at other Poor-Law infirmaries, such as Camberwell and Southwark, it would appear that the above salaries are still hardly commensurate with the arduous work which now devolves upon the junior medical officers. The moral is that as long as Boards of Guardians offer inadequate salaries they are not likely to improve the prospect of filling vacancies with qualified practitioners of the first class.

### INCREASE OF SALARY FOR AN INFIRMARY SUPERINTENDENT.

After twenty-four years the Woolwich Board of Guardians have decided to increase the salary of Dr. W. E. Boulter, medical superintendent of their infirmary, to £350 a year. At various times his remuneration has been rearranged and extra duties placed upon him, yet despite these the salary remained at the same figure as the Board fixed it twenty-four years ago. If the war has done nothing else, it has at least awakened Boards of Guardians to the fact that, in a great many instances, they have been underpaying their medical staff, and that, if they desire to retain their services, they must increase their salaries. In many cases the superintendent of an infirmary has been content to remain at a low salary because he has become thoroughly imbued with a love for the work and a desire to secure progress in it, thus rendering a public service to the community. This view of the duties, whilst being one that commands esteem and respect, has perhaps had an ill-effect on others, who, equally possessed of the same philanthropic spirit, have realised from the first that their work has been considerably underpaid. Perhaps if less modesty was displayed by many of the superintendents of the Metropolitan infirmaries there would be a general rise in the appreciation of their services, not only by the Boards of Guardians and the public, but by that most conservative authority, the Local Government Board.

### THE WASTE OF TOBACCO.

The cry of wasteful charity is always raised after a war has been in progress for some time, and generally with truth. Huge demands are suddenly made, and funds are generously subscribed before an organisation for dealing with them has been devised, still less got into working order. A specific instance of waste in connection with hospital work was given in the *Times* last week by Dr. F. M. Sandwith, chairman of the County of London Branch of the British Red Cross Society. In quoting examples of overlapping from an article written by Mr. J. Hossack in the *British Medical Journal*, Dr. Sandwith alludes to the writer's complaints of waste in the matter of garments supplied to the hospitals at St. Malo. He also quotes Mr. Hossack as complaining of "the unnecessary quantity of tobacco and cigarettes sent by all sorts and conditions of people and societies." Dr. Sandwith adds, with pointed brevity: "I have reason to believe that his experience is by no means unique." There can be no doubt that when the history of the war, including its minor abuses, comes to be written, the inordinate excess of tobacco-smoking will be presented to posterity as one of the astonishing features of charitable effort. The men, and in certain of the Voluntary Aid hospitals the "nurses," are often encouraged to smoke without restraint. The result is, what can only be expected, there is much waste, often a bad effect on the health from "smoker's heart," and an effect, no less bad, on the tone of those institutions where the "nurses" participate with the patients in the tobacco habit. It is not a case of reasonable indulgence. It is a case of excess; and in a time when the appeal for national economy is being made in the Press and on platforms, the undue consumption of tobacco, an import which could well be reduced in quantity, should be checked both inside and outside the hospitals. In the case of the drink traffic some classes consume markedly more than others. In the case of the tobacco traffic, all classes are consumers. Is that why the need for economy in its consumption has not been pressed?

### HEALTH OF THE TROOPS IN THE DARDANELLES.

In reply to Mr. Joynson-Hicks, Mr. Tennant, Under-Secretary of State for War, gave in the House of Commons last week some details concerning the health of the troops in the Dardanelles. There was, said Mr. Tennant, a certain amount of enteric fever and dysentery among our troops in the Dardanelles. The Director-General of the Army Medical Service had received from the General Officer Commanding in Egypt a letter in which he said: "I wish you could see what is being done. There were difficulties which were enormous. There was some want of organisation; but it is all now remedied or in process of being remedied. I have gone on the principle of asking you for everything I want; and here I have spared no expense to improvise. With regard to doctors and nurses, you have always responded, and we have never really been in want." It will be remembered that certain allegations have been current concerning a lack of preparation in Egypt for the reception, accommodation, and treatment of the wounded received from the Gallipoli Peninsula.

### PATENT MEDICINES AND GIFTS TO HOSPITALS.

By what may be called a beneficent irony of fate, one of the most important series of charitable bequests to hospitals is contained in the will of a gentleman who amassed a fortune from the sale of a proprietary medicine. The testator is the late Mr. J. C. Eno, of Woodhall, Dulwich, the proprietor of "Eno's Fruit Salt," who died on May 11 last, at the age of eighty-seven, and left estate the net personalty of which amounts to £1,585,823. Mr. Eno has bequeathed £50,000 to Guy's Hospital, half of which is earmarked for general purposes, and half for the re-endowment fund. His other bequests include £40,000 to the Royal Victoria Infirmary, Newcastle-upon-Tyne; and £20,000 to the London Lock Hospital and Rescue Home. This is the fourth millionaire estate of the current financial year, which began on April 1, and the fifth since January 1. Mr. Eno's career, and he is described as a manufacturing chemist, is interesting in several ways. He is understood to have taken an active interest in the drafting of the firm's advertisements, which have been characterised for the amount of matter contained in them, and for the literary and philosophic quotations in which they have often abounded. How far he was a sharer in the advertising ideals of the founder of the firm of Pears is uncertain. But the nature of his firm's advertisements suggests that, just as the soap manufacturer secured the services of Millais in the endeavour to raise the tone of display advertisements by the judicious employment of artistic skill, so

Mr. Eno may have endeavoured to raise the tone of advertising "literature" by copious quotations from philosophic and poetic masterpieces. At all events, these lengthy screeds seem to have been read and appreciated, and one of the results has been the large bequests which hospitals are now receiving under his will. Readers of "Tono-Bungay" will remember that the proprietor of the patent medicine, whose rise and fall are described in its pages, used to defend his activities on the ground that "he minted faith" for countless sufferers!

### A STRANGE BEQUEST TO A DOCTOR.

Precautions against premature burial have been many and strange, but few can have been more drastic than that provided for in the will of the late Miss Emily Harriet Ripley, of Carshalton, Surrey. Her bequests included one to a medical practitioner, Dr. Stanley Bousfield, to whom she bequeathed a legacy of £20 upon the condition that he should see that her throat was cut from ear to ear before burial. It is noteworthy that while the number of those who fear premature burial is relatively small, those who are subject to it become almost obsessed, and take, or arrange for, elaborate surgical precautions. The view that cremation is the best and simplest precaution, if only on account of the careful death certification which it entails, seems too simple a solution to commend itself to those who are obsessed by the risk of the possibility of premature burial.

### THE RED CROSS MOTOR LAUNCH.

A statement has been published by Lieutenant-Colonel P. J. Blackham, General Secretary, St. John Ambulance Association in India, summarising the work of the Red Cross now being undertaken in India. After pointing out that the whole organisation is in the hands of the Indian Council of the St. John Ambulance Association—the Red Cross Society of India—he proceeds to detail for what duties it is responsible, apart from providing for the needs of the Indian Expeditionary Force. A fleet of motor ambulances for service with the war hospitals both at the Front and in India has been raised. Motor launches have been provided for employment in the Persian Gulf and on the coast of East Africa. Nearly two wards have been endowed in the war hospital founded by the Order of St. John of Jerusalem for the armies in the field. Moreover, a St. John Ambulance War Hospital has been established in India, and offered to the Government of India for special cases of wounded officers and men from oversea. It will be organised and maintained by the Indian Council, and will be conducted in association with the X-ray Institute at Dehra Dun. This is a fine record which might be added to, for the Indian Red Cross has also provided comforts for the troops (including religious books for the Sikhs and Mahomedans in the military hospitals in Europe) and gifts of money and materials to the Red Cross Societies of the Allies. It is but the latest instance of the solidarity of the Empire in the common cause.

### AN UNFORTUNATE MISTAKE AND ITS LESSON.

The need for scrupulous care in the handling of drugs, even if the drugs are not poisons in the ordinary sense, is emphasised by the report of an inquest which has recently appeared in the newspapers; and incidentally this case shows how necessary it is that in public institutions—which often distribute more drugs than ordinary chemists' shops—the dispensary should be under the control of a trained person. According to the report a woman, who was accustomed to taking an occasional dose of sulphate of soda, was supplied at a drug store with tartaric acid by mistake, the error being due, it appears, to some rearrangement of the bottles. She took a dose of the tartaric acid, was taken ill shortly afterwards, and died within a few days. Medical evidence showed that death was due to heart disease and congestion of the lungs and irritation of the stomach; Dr. Spilsbury stated that tartaric acid, if taken in excess, would set up irritation of the stomach and he was unable to exclude that from the cause of death. The case is an extremely unusual one, but the moral is obvious.

### AN ARMY PHARMACEUTICAL SERVICE.

In carrying out the duties (alluded to in a recent paragraph in these Notes) which were lately detailed by Major Peck, the pharmacist would certainly need the authority of commissioned rank. At present there are no signs that this dream of an Army Pharmaceutical Corps as a separate establishment of His Majesty's Army will come true, but, as has been shown in The Hospital, there are very good reasons why the Pharmaceutical Service of the Army should be thoroughly reorganised.

### A HERO AT WESTMINSTER HOSPITAL.

Twenty sick and wounded soldiers who have been serving in the Dardanelles were admitted into Westminster Hospital last Saturday, and among them was Sergt. J. L. Dewar, 4th Battalion Royal Scots (Queen's Edinburgh Rifles), who won the King's Prize last year at Bisley after a tie with Private Fulton, of the Queen's Westminster Rifles. Sergt. Dewar, who is suffering from sunstroke, has a story of courage to his credit which is well worth recording. Two enemy snipers were causing much annoyance to our troops, and Sergt. Dewar determined that he would try to stop them. He, therefore, went out and not only succeeded in picking off the two snipers but also secured their rifles, an act of daring which led to his being discovered by the enemy, and made it impossible for him to return to the British lines. For four hours he lay flat on his face, the bullets whistling around him and the sun beating on the back of his neck. He was ultimately rescued in a state of insensibility. His nervous system is naturally affected, but fortunately he was not wounded.

### A CHILDREN'S WARD FOR SOLDIERS.

The Montagu Hospital, Mexborough, which ordinarily possesses thirty-two beds, has now had formally opened a new children's ward. This has been built at a cost, in round figures, of £1,600. In declaring it open the other day Mr. John Clayton, chairman of the board of management, pointed out that the ward was not, in the first instance, to be devoted to child patients, but that it is to form part of the accommodation which the hospital is placing at the disposal of the 3rd Northern Base Hospital as a hostel for wounded soldiers. It is now just over three years since a fund was started to provide for the new ward, which is furnished with a sun balcony, having a collapsible front, thanks to the generosity of the Hon. Mrs. H. Lindley Wood. This gift is in memory of her first husband, Mr. James Montagu, the founder of the original Montagu Cottage Hospital. The architects are Messrs. G. Moxon and Son.

### MEDICAL STUDENTS AND THE NEW MASTER OF DOWNING.

A meeting of the professors of Downing College, Cambridge, has been lately held for the appointment of a master in succession to the late Professor F. Howard Marsh, M.A., F.R.C.S., D.Sc. Mr. Albert Charles Seward, M.A., F.R.S., Hon. Sc.D. Dublin, Fellow of St. John's College, Cambridge, Honorary Fellow of Emmanuel College, Cambridge, and Professor of Botany in the University, was appointed. Professor Seward, who is fifty-two, was educated in his early years at the Grammar School at Lancaster. Although his chief research has been in connection with the geological aspect of botany, he is well known to medical students in their studies for the Natural Science Tripos. Professor Seward's many friends will congratulate him upon his election to the mastership of Downing, and he will be welcomed by all members of the College.

### FARM WORK] FOR EPILEPTICS.

The Home for Epileptics, Maghull, provides an interesting example of what can be done by patients of this class in agricultural pursuits. There are 353 patients, every one of whom, who is capable of such work, undertakes a share in the work of the colony. While farming and gardening provide occupations for the men, the women are employed at laundry work, basket making, sewing, and domestic duties. How practical the work is may be judged from the fact that the accounts give figures showing a substantial profit. Indeed, the general accounts are stated to benefit by a sum of £432 from this source. Although the Home itself is the principal consumer of the produce of the farm, goods to the value of £927 have been disposed of in the general market during the past year. The Board of Education has increased its grant from some £300 to £600. Education, indeed, plays an important part in the organisation of the colony; a continuation school exists, and classes are held not only in the "three R's," but also in painting, singing, physical culture, and even wood-carving, metal-work, and, of course, needlework and sewing. The cost of maintenance is understood to be borne mainly by the patients and their friends, while certain generous donors have given the various Homes wherein the patients are accommodated. Of course, in considering the productivity of the patients it is always necessary to take into account the cost of supervision, without which their work would not be done at all. This is not easy to estimate, but there is abundant evidence to show that with advantage to themselves they may yet become, if in a limited sense, actual producers.

### THE SEELY MEMORIAL AT NOTTINGHAM.

We were compelled to hold over from our last issue an account of the decision of the monthly board of Nottingham General Hospital to place a permanent memorial within the hospital to the late Sir Charles Seely, Bart. Mr. Frederick Acton, the chairman, together with the hospital's president, Sir Jesse Boot, and the vice-chairman, are inviting subscriptions, and the form of the memorial will probably depend upon the amount subscribed. The late Sir Charles's son, the new baronet, who has been elected a member of the monthly board, is a colliery owner, and was formerly Member of Parliament for Lincoln. Colonel Seely, formerly Secretary of State for War, is the youngest son of the late baronet. The Nottingham General Hospital, it will thus be seen, has intimate associations with the war, and the treasurer of the hospital, Mr. Abel Bertram Smith, is now in Egypt with his regiment.

### BANK HOLIDAY AND THE WEEK'S DRUG MARKET.

Since last report appeared the market has been quiet owing to the intervention of Bank Holiday, but it is not likely that the holiday feeling will be so prolonged as at normal times. The general tendency of prices is still in an upward direction, but there are several exceptions. There is practically no change in the position of cod-liver oil and the high prices are well maintained, although buyers are holding off the market. There has been no further advance in the price of Epsom salts, but the scarcity continues. The value of olive oil is slowly advancing. Zinc oxide, zinc sulphate, and other medicinal salts of zinc are dearer in sympathy with the rise in price of the metal. Belladonna leaves, which are still scarce, are fetching high prices. Gelatine is dearer. An advance in the price of glycerine is considered probable. Gum acacia is dearer. The high prices of salicylic acid and salicylate of soda are well maintained. The available supply of myrrh is short and prices have a tendency to advance. Gamboge is dearer. Liquorice root is advancing in price. Ergot of rye is cheaper as a result of more plentiful supplies. Buchu leaves have an easier tendency in price. The value of santonin is not quite so firmly maintained.

### TO OUR READERS.

Contributions are specially invited from any of our readers to these columns. They should deal with topical subjects and news. They must be authenticated for the information of the Editor only. The minimum payment if published is 5s. There is no hard-and-fast rule as to space, but notes of about twenty lines in length are preferred.

